# How Perturbated Metabolites in Diabetes Mellitus Affect the Pathogenesis of Hypertension?

**DOI:** 10.3389/fphys.2021.705588

**Published:** 2021-08-18

**Authors:** Zhangchi Ning, Zhiqian Song, Chun Wang, Shitao Peng, Xiaoying Wan, Zhenli Liu, Aiping Lu

**Affiliations:** ^1^Institute of Basic Theory for Chinese Medicine, China Academy of Chinese Medical Sciences, Beijing, China; ^2^School of Chinese Medicine, Hong Kong Baptist University, Hong Kong, China

**Keywords:** diabetes mellitus, the pathogenesis of hypertension, perturbated metabolites, metabolic function, connected metabolic mechanism

## Abstract

The presence of hypertension (HTN) in type 2 diabetes mellitus (DM) is a common phenomenon in more than half of the diabetic patients. Since HTN constitutes a predictor of vascular complications and cardiovascular disease in type 2 DM patients, it is of significance to understand the molecular and cellular mechanisms of type 2 DM binding to HTN. This review attempts to understand the mechanism via the perspective of the metabolites. It reviewed the metabolic perturbations, the biological function of perturbated metabolites in two diseases, and the mechanism underlying metabolic perturbation that contributed to the connection of type 2 DM and HTN. DM-associated metabolic perturbations may be involved in the pathogenesis of HTN potentially in insulin, angiotensin II, sympathetic nervous system, and the energy reprogramming to address how perturbated metabolites in type 2 DM affect the pathogenesis of HTN. The recent integration of the metabolism field with microbiology and immunology may provide a wider perspective. Metabolism affects immune function and supports immune cell differentiation by the switch of energy. The diverse metabolites produced by bacteria modified the biological process in the inflammatory response of chronic metabolic diseases either. The rapidly evolving metabolomics has enabled to have a better understanding of the process of diseases, which is an important tool for providing some insight into the investigation of diseases mechanism. Metabolites served as direct modulators of biological processes were believed to assess the pathological mechanisms involved in diseases.

## Introduction

Diabetes mellitus (DM) is an endocrine metabolic disease with an increasing prevalence all over the world. According to data released by the International Diabetes Federation, the number of DM patients is predicted to rise to 552 million by the year 2030. Type 1 and type 2 are the two main types, with type 2 DM accounting for the majority (>85%) of total DM prevalence (Forouhi and Wareham, 2014). Hypertension (HTN) is a crucial risk factor for type 2 DM-associated vascular complications. Vascular process whereby type 2 DM and HTN predispose to cardiovascular diseases. When combined with DM, HTN has been shown to predict and promote increased risk for cardiovascular diseases, which is the major cause of mortality in patients with type 2 DM (Petrie et al., 2018). Furthermore, up to 75% of diabetic cardiovascular diseases may be attributable to HTN (Sowers et al., 2001). There is a clinical impression that HTN occurs more frequently among diabetic patients than in the healthy population. It was proved to present in more than 50% of patients with type 2 DM. It is closely related to the occurrence and development of diabetic microvascular and macrovascular diseases. Indeed, DM and HTN were four times more likely to develop cardiovascular disease than non-diabetic controls with normal blood pressure. They also increased the incidence of other diabetes complications, such as kidney disease and retinopathy (Lastra et al., 2014). Hence, it is urgent to understand the molecular and cellular mechanisms of the combination of DM and HTN.

The rapidly evolving field of metabolomics is aiming at a comprehensive measurement of all endogenous metabolisms in biological systems (Acevedo-Calado et al., 2017). It represents both the upstream input from the environment and the downstream output of the genome (Wishart, 2016). Panels of multiple biomarkers reflecting their specificity and potentials for prediction have been developed (Savolainen et al., 2017a). In recent years, the value of metabolomics has been redefined from biomarker recognition tools as a technology for active drivers of biological processes (Johnson et al., 2016). The search for genes that are associated with metabolite levels, or whose products catalyze reactions, including disease-related metabolites and pathways, may explain the way these metabolic phenotypes are regulated (Zhang et al., 2018).

Moreover, metabolic research could also make it possible to understand the effects of specific microbes on specific metabolites better. Growing evidence accumulated that gut microbiota may not be overlooked in the pathogenesis of diseases. The gut microbiota affects the host by producing metabolites systematically. Small-molecule metabolites are one of the contributions of gut bacteria to host biology (Dodd et al., 2017). Metabolomics is a promising technology to contribute to the disease mechanism investigation since metabolites were served as direct modulators of biological processes and active entities in a biological process.

This review attempts to understand the mechanism of the combination of type 2 DM and HTN *via* the perspective of the metabolites. Firstly, it focuses on the metabolic perturbations of type 2 DM and HTN. After summarizing the biological function of perturbated metabolites, the paper associated the perturbated metabolites with the mechanisms in type 2 DM and HTN. Finally, the review reveals the effect of type 2 DM-associated metabolic perturbations on the pathogenesis of HTN.

## Metabolic Perturbations in Type 2 DM and HTN

Metabolites are substrates or products of metabolic pathways. The changes in the metabolome directly reflect the gene expression, physiological status, and environmental effects on the biological system (Monteiro et al., 2013). Metabolic perturbations have been studied to diagnose and prediction of diseases by biomarkers. Over the past 15years, advances in metabolomics techniques, together with improvements in bioinformatics and mathematical modeling approaches, have provided the scientific community with tools to describe DM and HTN metabolomics. Three general techniques have become the main tools for metabolomics data acquisition: liquid chromatography-mass spectrometry (LC-MS), gas chromatography-mass spectrometry (GC-MS), and nuclear magnetic resonance (NMR). Metabolites are typically separated using gas chromatography (GC) and liquid chromatography (LC) before MS, and compounds are identified by mass and fragmentation patterns. However, there is no need to separate metabolites in NMR spectroscopy instead of measuring all metabolites at once. The use of multiple technologies was proved to broaden the level of metabolite coverage greatly. Metabolomics raw data processing involves noise reduction, spectrum deconvolution, peak detection and integration, chromatogram alignment, compound identification, and quantification (Shulaev, 2006). It is of great significance to perform statistical analysis on the processed data, reduce the number of variables, and obtain irrelevant features in the data. It can be best achieved either through unsupervised significance algorithms, such as principal component analysis (PCA), or through supervised methods, including *t*-test, χ^2^-tests, ANOVA, orthogonal partial least square discriminant analysis (OPLS-DA), and partial least squares (PLS; Lazar et al., 2015). The numbers of metabolites and metabolic classes have garnered much attention as biomarkers in the field of DM and HTN investigations. Validated biomarkers become alter markers that can substitute for clinical endpoints to monitor disease progression. An overview of the observed metabolic changes of type 2 DM and HTN is shown in Table 1. The comparison and correlation of the perturbated metabolites in type 2 DM and HTN were performed. The perturbated metabolites in common, perturbated metabolites in type 2 DM, and the perturbated metabolites in HTN were listed individually.

**Table 1:** Summary of metabolic perturbations of DM and HTN.

Type of metabolites	Metabolic perturbations of DM	Metabolic perturbations of HTN	Common metabolic perturbations of both DM and HTN	Multivariate statistical method	References
Amino acid	N6-acetyllysine; N-trimethy-l5-aminovalerate; 2-oxoarglinine; homocitrulline; N-acetylcitrulline; N-delta-acetylornithine; N2, N5-diacetylornithine; isoleucine; leucine; threonine; tryptophan; tyrosine; γ-glutamyl-leucine; N,N-dimethylglycine; tau-methylhistidine; asparagine; n-alpha-acetyllysine; hydroxyproline; aspartate; proline; dimethylglycine; 2-aminoadipic acid; β-alanine; and histidine.	L-arginine; phenyl propionic acid; 3-hydroxyproline; β-aminoisobutyric acid; 4-hydroxy-proline; and N-acetylornithine	Arginine; valine; Alanine; tyrosine; glycine; lysine; glutamine; threonine; tryptophan; phenylalanine; citrulline; methionine; ornithine; serine; glutamate; and cystine	Bonferroni-corrected threshold, Pearson correlation coefficients, Wilcoxon signed-rank, independent-sample *t*-test, χ^2^-tests, PCA, OPLS-DA on Pareto scaled, Pearson correlation coefficients, Partial least-squares PLS-DA, Logistic regression models, PLS, McNemar’s test, Fisher’s exact test, Mann–Whitney U-test, orthogonal signal correction (OSC), and random forest analysis.	Lu et al., 2008; Wang et al., 2011; Ho et al., 2013; Würtz et al., 2013; Cooper-DeHoff et al., 2014; Pena et al., 2014; Friedrich et al., 2015; Palmer et al., 2015; Yokoi et al., 2015; Fall et al., 2016; Nikolic et al., 2016; Hernández-Alvarez et al., 2017; Kappel et al., 2017
Energy-related metabolites	6-oxopiperidine-2-carboxylate	D-methylglucopyranoside		PCA, PLS-DA, OSC, paired Student *t*-test, independent-sample *t*-test, Chi-squared tests, ANOVA, orthogonal projection to latent structure discriminant analysis (OPLS-DA), nonparametric Mann–Whitney, and nonparametric Kruskal-Wallis test.	Brindle et al., 2003; Akira et al., 2008; Lu et al., 2008; Fiehn et al., 2010; Liu et al., 2011; Guan et al., 2013; Ho et al., 2013; Li et al., 2013; Filla et al., 2014; Pena et al., 2014; Grapov et al., 2015; Rotroff et al., 2015; van Deventer et al., 2015; Wu et al., 2015; Allalou et al., 2016; LauraGonzalez-Calero et al., 2016; Nikolic et al., 2016; Wang et al., 2016; Yu et al., 2016; Kappel et al., 2017; Martin-Lorenzo et al., 2017
Lipids and fatty acids	SMs; PCs; palmitoleic acid; lysoPC/PC (O-16:1/0:0); monoacylglycerol (18:2), CerPE (38:2); SM (d18:2/18:1); SM (d18:2/18:1); nitric oxide-derived saccharic acid; arachidonic acid; linoleoylglycerophosphocholine; diacyl-phosphatidylcholines C32:1, C36:1, C38:3, C40:5; sphingomyelin C16:1; acyl-alkyl-phosphatidylcholines C34:3, C40:6, C42:5, C44:4, and C44:5; sphingomyelin C16:1; acyl-alkyl-phosphatidylcholines C34:3, C40:6, C42:5, C44:4, and C44:5; lysophosphatidylcholine C18:2; triacylglycerols; cholesterol esters; linoleic acid (18:2 n-6); palmitic acid (16:0); onounsaturated palmitoleic (16:1 n-7); oleic (18:1 n-9) acids; PE (C16:0/C22:6); PE (C18:0/C20:4); stearic acid; Linoleic acid; palmitic acid; eicosapentaenoic acid; and 3-carboxy-4-methyl-5-propyl-2-furanpropanoic oleic acid	13-OxoODE; 9-OxoODE; sphinganine 1-phosphate; sphinganin; thromboxane; PA (16:0/16:0); 9(S)-HPODE; LPA (0:0/18:0); 9,12,13-TriHOME; phytosphingosine; 12(S)-HPETE; HETE; HDoHE; hexacosahexaneoic acid; behenic acid; 1-stearoylglycerol; hexadecanoid acid; stearic acid; ceramide; acyl-alkyl-phosphatidylcholines C42:4; C44:3; diacyl-phosphatidylcholine C38:4; C38:3; monohexosylceramides; and phosphatidylinositols	PCs; diacylglycerols; lysoPCs; and linoleic acid	ANOVA, Benjamini-Hochberg procedure, PCA, PLS-DA, *t*-test, χ^2^-tests, and least absolute shrinkage and selection operator (LASSO) regression.	Kotronen et al., 2009; Mäkinen et al., 2012; Floegel et al., 2013; Dietrich et al., 2016; Fall et al., 2016; Yu et al., 2016; de Mello et al., 2017; Liu et al., 2017a,c, 2018; Bujak et al., 2018; Tian et al., 2018
Carnitine	Tiglylcarnitine; benzoylcarnitine; 3-methyladipoylcarnitine; octanoylcarnitine; cis-4-decenoylcarnitine; decanoylcarnitine; arachidonoylcarnitine (C20:4); 3-hydrixybutyrylcarnitine; acylcarnitine; 2-methylbutyroylcarnitine; palmitoylcarnitine; carnitine; butenoylcarnitine; and 3-hydrixybutyrylcarnitine	\	\	Pearson correlation coefficients, Wilcoxon signed-rank, independent-sample *t*-test, χ^2^-tests, principal component analysis (PCA), and orthogonal partial least square discriminant analysis (OPLS-DA)	Kappel et al., 2017
Bile acid	deoxycholic acid and cholate; deoxycholates	Hydroxyoxocholanoic acid	\	PCA, PLS-DA, OSC, paired Student *t*-test, independent-sample *t*-test, and Chi-squared tests	Ho et al., 2013; Fall et al., 2016; Bujak et al., 2018
Others	Pipecolate; urea; cortisol; trimethylamine N-oxide; allantoin; amines; carbonyls; cholate; cystathionine; indole propionate; uridine; trimethylamine; quinolinate; urate; acetoacetate; methylamine; glyoxal hydroimidazolone; 3-deoxyglucosone hydroimidazolone; and alkylresorcinols C17 and C19	17a-hydroxypregnenolone; 5,6-dihydroxyprostaglandin F1a; decanamide; dodecanamide; 1-hexadecanol; erythritol; alpha-1 acid glycoproteins; choline; sitosterol; adenine; uracil; glycerate; 3-ureidopropionate; 3-hydrixybutyrate; guanidinoacetate; hexadecanedioate; dicarboxylic acid; epiandrosterone sulfate; 5α-androstan-3β; 17β-diol disulgate; androsterone sulfate; 4-hydroxyhippuric; hippurate; N-methylnicotinate; and creatine	Formate; kynurenine; xanthine; pantothenate; creatinine; 5-hydroxy indole acetic acid; tocopherol; and betaine	ANOVA, Benjamini-Hochberg procedure, PCA, PLS-DA, *t*-test, χ^2^-tests, least absolute shrinkage and selection operator (LASSO) regression, and ANOVA	Akira et al., 2005; Holmes et al., 2008; Ferrannini et al., 2013; Guan et al., 2013; Ho et al., 2013; Menni et al., 2013; Zheng et al., 2013; Grapov et al., 2015; Rotroff et al., 2015; Yokoi et al., 2015; Fall et al., 2016; Wang et al., 2016; de Mello et al., 2017; Kappel et al., 2017; Murfitt et al., 2017; Savolainen et al., 2017b; Bujak et al., 2018; Tian et al., 2018

**Figure 1:**
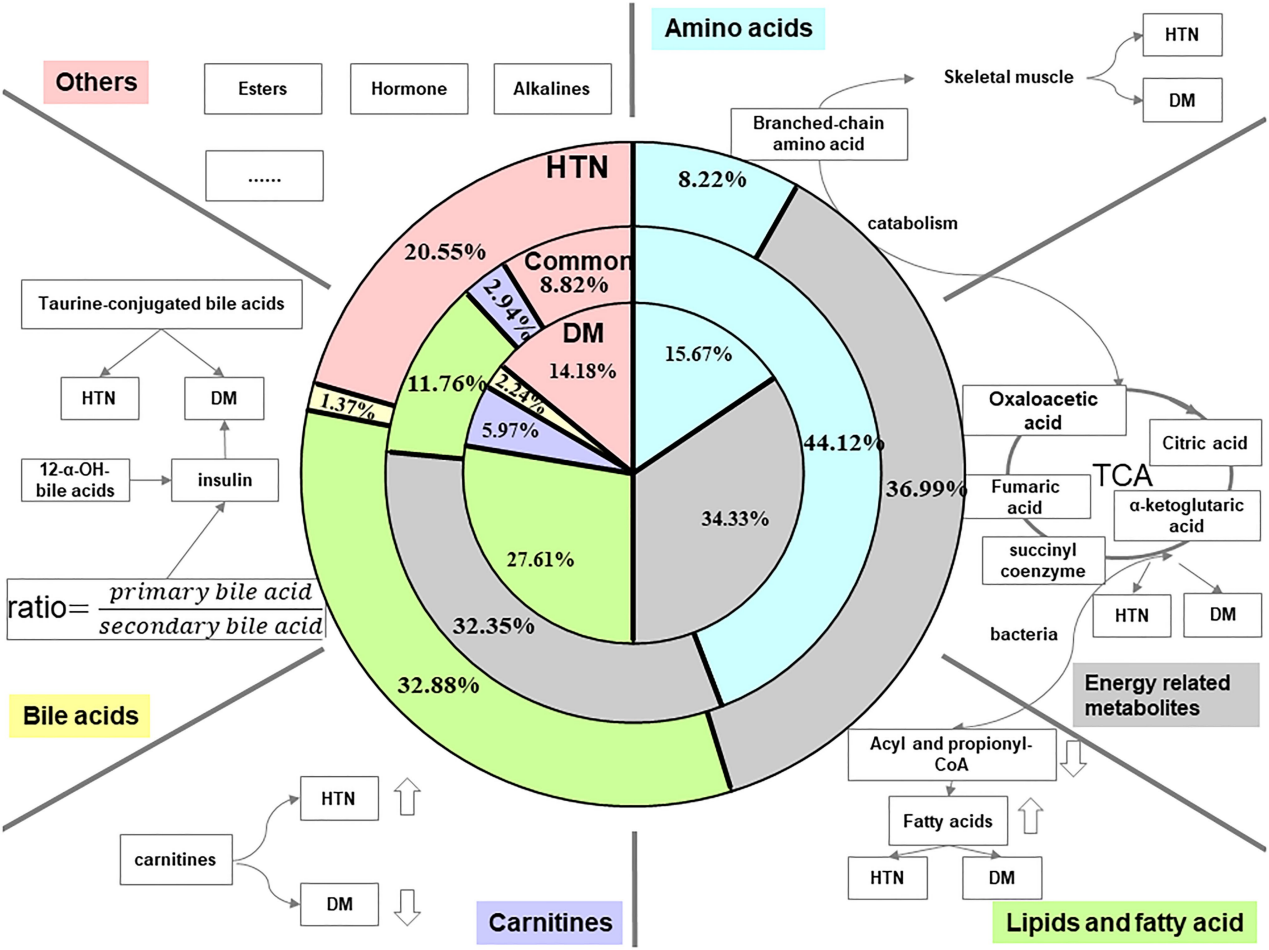
Summary of related metabolites of DM and HTN. The three pie charts, respectively, illustrate data regarding the distribution of different kinds of metabolites in HTN, common, and DM. The blue color represents the proportion of amino acids in the total reported metabolic perturbations. Amino acids occupy 8.22, 15.67, and 44.12% of perturbated metabolites in HTN, DM, and the common biomarkers of both DM and HTN, respectively. The gray color indicated the percentage of energy-related metabolites. The proportion of energy-related metabolites is almost evenly in HTN, common, and DM. The green color shows the partition of lipids and fatty acids. The lipids and fatty acids section consumed a little bit lower of 11.76% in the common perturbation metabolites, while the rest two have an almost equal share of 30% approximately. The purple and yellow indicated the makeup of carnitine and bile acids in HTN, DM, and the conventional biomarkers of both DM and HTN. Compared to other metabolites, these two kinds of perturbation had the smallest number. The pink color represents other sorts of perturbations in HTN, DM, and the common. They share less than a quarter of metabolic perturbation in HTN and make up 14.18% in DM and 8.82% in common.

The metabolites, including amino acids, energy-related metabolites, lipids and fatty acids, carnitines, and bile acids were associated with type 2 DM and HTN. According to Table 1, we calculated proportions of different kinds of perturbated metabolites in the whole panel of metabolic markers of DM, HTN, and both of the two diseases individually and showed the ratios of each metabolic marker classes in DM, HTN, and the common of two diseases in the pie chart of Figure 1.

Amino acids – Based on the review of the relevant literature, we listed the disturbances of amino acids in DM and HTN in Table 1 and pictured the blue pie charts to represent the ratio of amino acids in the whole panel of metabolic markers in Figure 1. The level of branched-chain amino acids (BCAAs; valine, leucine, and isoleucine) and aromatic amino acids (AAAs; tyrosine, phenylalanine, and tryptophan) was increasing in DM adults (Walford et al., 2013). Although most research is conducted in European populations, an analysis in Chinese and Asian-Indian men showed that the associated decrease in glycine was consistent with the increase in valine and leucine levels with multiple presentations of the development of type 2 DM and insulin resistance (Huffman et al., 2011). However, their role in the few available pediatric studies was controversial, with lower levels of BCAA (Michaliszyn et al., 2012; Mihalik et al., 2012; Frohnert and Rewers, 2016). A case-cohort study showed that the increased plasma levels of BCAAs/AAAs were associated with higher type 2 diabetes risk (Ruiz-Canela et al., 2018). Glycine and glutamine are reduced in insulin resistance (Sekhar et al., 2011; Stančáková et al., 2012). The potential role of BCAAs and AAAs with incident HTN is increasingly recognized. High plasma concentrations of BCAAs and AAAs are proved to be associated with an increased risk of newly developed HTN (Teymoori et al., 2018; Flores-Guerrero et al., 2019). According to the clinical implications, BCAAs were associated with the incident of type 2 DM. They also seem on the pathway from DM to cardiovascular diseases for the attenuation of the association of BCAA and cardiovascular patients without type 2 DM. However, it is still too early to tell whether BCAAs are the marker of risk for DM or cardiovascular diseases in DM.

Energy-related metabolites – The gray pie charts represent the ratio of energy-related metabolites in the whole panel of metabolic markers in Figure 1. The energy metabolism abnormalities are commonly associated with DM and HTN. The two diseases are related to gluconeogenesis, glycolysis, pentose phosphate, and tricarboxylic acid (TCA) cycle (Iemitsu et al., 2003; Ho et al., 2013). Hexose sugars are primarily accounted for by disease-defining increasing glucose and positively associated with DM. And the elevations of fructose, mannose, sorbitol, lactate, and malate have been proved to be associated with DM (Fiehn et al., 2010; Hwang et al., 2010; Menni et al., 2013). α-ketoglutarate, fumarate, and succinate levels in urine are decreased in DM patients (Salek et al., 2007).

Lipids and fatty acids – The green pie charts represent the ratio of lipids and fatty acids in the whole panel of metabolic markers in Figure 1. Given the significance of obesity as one of the risk factors for type 2 DM and HTN, it is not surprising that both DM and HTN patients have increased fat-derived metabolites. Phosphatidylcholine (PC), lysophosphatidylcholine (LPC), phosphatidylethanolamine (PE), and diacylglycerol (DAG) increased the risk of DM (Floegel et al., 2013). Conversely, a major class of sphingolipids, sphingomyelins (SM), and ceramide (Cer) was linked with a decreased risk of DM (Fall et al., 2016). Thromboxane, linoleic, phosphatidic acid (PA), lysophosphatidic acid (LPA), and phytosphingosine were increased, while sphinganine decreased in the HTN group (Tian et al., 2018). More and more evidence pointed to a mental disorder sphingolipid pathway trigger HTN. Hydroxyoctadecadienoic acids (HODEs) are related to HTN, the generation of which is increased where oxidative stress is increased. 9-HODE and 13-HODE are generated nonenzymatically in atherosclerosis. Increased HODE levels were proved to contribute to HTN and atherosclerosis progression (Vangaveti et al., 2010). Richard et al. highlight the discovery of 20-hydroxyeicosatetraenoic acid (20-HETE) in the regulation of renal function, vascular tone, and the development of HTN (Roman and Fan, 2018). The increasing levels of 12-HETE and 12-hydroperoxyeicosatetraenoic acid (12-HPETE) in HTN patients suggested roles for these metabolites in HTN (González-Núñez et al., 2000). The perturbed lipids in DM and HTN are listed in Table 1.

Carnitines – The purple pie charts represent the ratio of carnitines in the whole panel of metabolic markers in Figure 1. Propionyl carnitine, α-methyl butyl carnitine, and isovaleryl carnitine were elevated in type 2 DM (Pena et al., 2014; Qiu et al., 2016). The decrease of short-chain acylcarnitines and long-chain acylcarnitines, including C14:1 and C14:2, was observed in HTN patients (Bai et al., 2018).

Bile acids (BAs) – The yellow pie charts represent the ratio of carnitines in the whole panel of metabolic markers in Figure 1. In healthy subjects, insulin resistance increased by 12α-hydroxylated BAs. The ratios of non-12α-hydroxylated and 12α-hydroxylate d BAs are related to the main characteristics of insulin resistance (Hausler et al., 2013). Marlene et al. revealed that total taurine-conjugated BA concentration was higher in DM patients (Wewalka et al., 2014). A systematic evaluation of bile acid kinetics revealed that the contribution of specific types of bile acids varies in type 2 DM even though there was no difference in the size of the total BA pool. The level of deoxycholic acid was increased, and chenodeoxycholic acid was decreased (Brufau et al., 2010). Another study showed that lower cholic acid and higher plasma levels of deoxycholic acid occurred in diabetic patients compared to controls (Suhre et al., 2010).

Amino acids, energy-related metabolites, lipids, and fatty acids made up the biggest percentage of the total disturbed metabolites compared with BAs and other metabolites. They may play a significant role in the pathogenesis of HTN in DM. Hence, the biological function of these metabolites should be illustrated. It also can be found that the commonality of DM and HTN among metabolites is limited. To reveal how they are different or more interestingly amalgamated in both disease phenotypes, which leads to an intermediate or tertiary state that the patient’s metabolic physiology, the biological mechanism should also be discussed.

## The Biological Function of Perturbated Metabolites in Type 2 DM and HTN

The metabolic biomarkers served as mechanistic discoveries. Metabolites modulate biological processes and phenotypes directly (Johnson et al., 2016). Direct modulation was associated with alterations in host metabolism, mainly through immune systems and hormone secretion (Li et al., 2018a). The metabolic potential of the gut microbiota is another contributing element in the development of DM and HTN (Kitai and Tang, 2017), with metabolites acting on distant target organs like the human host’s endocrine organ. The representative biological functions of each class of metabolites are shown in Figure 1.

Amino acids – Studies of BCAAs, such as valine, leucine, and isoleucine supplementation in animals and humans, suggest that circulating amino acids may promote insulin resistance directly, possibly *via* disrupting insulin signaling in skeletal muscle. BCAA in combination with hyperinsulinemia exerts a large amount of secretory pressure on pancreatic accessory cells, resulting in dysfunction of accessory cells. BCAAs catabolism offered intermediates to the TCA cycle, which may boost energy production (O’Connell, 2013). Metabolism studies indicated that changes in TCA flux in diabetic patients (Guan et al., 2013; Li et al., 2013). The reduction of the tricarboxylic acid cycle was shown in DM and HTN (Brindle et al., 2003). Betaine is a tertiary amine dimethylglycine (DMG) produced by homocysteine remethylation to methionine. It is catalyzed by betaine-homocysteine methyltransferase (BHMT). DMG is catalyzed by DMG dehydrogenase (DMGDH) and sarcosine dehydrogenase in turn. Epidemiological data indicated that plasma levels of betaine correlate with key components of the metabolic syndrome in the opposite direction (Konstantinova et al., 2008). High plasma glycine levels are also thought to be associated with increased insulin sensitivity (Menni et al., 2013).

Metabolites involved in energy metabolism – Energy metabolism includes energy release and energy restoration, which provides a source of energy for the daily activity of cells. Mitochondrial is of significance in maintaining cellular energy metabolic homeostasis (Alston et al., 2017). The disequilibrium of mitochondrial functions in type 2 DM leads to the deficits of downstream in several parts, including cardiac output, skeletal muscle contraction, β-cell insulin production, and hepatocyte metabolism (Pinti et al., 2019). HTN, one of the cardiovascular diseases, is closely related to myocardial energy metabolism (Polak-Iwaniuk et al., 2019). Recent research proved that the improved skeletal muscle energy metabolism correlates to the recovery of β cell function and benefits the glucose control of type 2 patients (Tang et al., 2019). Fatty acids are the main fuel for the heart, while glucose and lactate serve the remaining need (Calvani et al., 2000). It is reported that the changes in glucose and fatty acids utilization appear in the diabetic heart fuel selection (Stanley et al., 1997). A downshifting of ketone body production and the breakdown and the tricarboxylic acid cycle was observed in insulin resistance (Heinonen et al., 2015). HTN shifts substrate preference toward increased glucose utilization in cardiac muscle. Citrate, pyruvate, malate, and adenosine monophosphate were the common metabolic markers for DM and HTN. When perfusion is reduced, there is an increase in the rate of glycolysis and a switch from lactate uptake to lactate production. During reperfusion, fatty acid oxidation quickly recovers. Otherwise, carbohydrates, such as hexoses, fructose, and gluconic acid, and nucleotides, such as adenosine, were the marker for DM and HTN, which linked to endothelial dysfunction, insulin resistance (Khitan and Kim, 2013).

Lipids and fatty acids – DM significantly decreased levels of acetyl- and propionyl-CoA means that it prevents the degradation of fatty acids, amino acids and ketone bodies. In contrast, an increase in free fatty acids (FFAs) occurred in HTN (Aa et al., 2010). FFAs regulate glucose counter-regulation by mediating hepatic glucose production (Fanelli et al., 1993). Lipolysis also mediates the delayed glucose-antiregulation pathway of growth hormone and cortisol stimulation. Hyperinsulinemic hypoglycemia increases the production of non-esterified fatty acids, indicating the role of lipids as alternative energy sources (Voss et al., 2017). The prevailing theory of lipid-induced hepatic insulin resistance is that lipid species accumulate due to oxidative damage to fatty acids, leading to the redirection of long-chain acyl-coenzyme A (LC-CoAs) to endoplasmic reticulum (ER) localization and cytosolic lipid species, such as triglycerides, diacylglycerols, and ceramides (Muoio and Newgard, 2008). Type 2 diabetes can be the result of the loss of or deficiency of beneficial functions, such as short-chain fatty acid produced by carbohydrate fermentation in the intestinal ecosystem (Zhao et al., 2018). Meanwhile, FFAs influence HTN (Aa et al., 2010). 12-HETE, transformed from arachidonic acid, acted as vasoconstrictors in the renal arteries, which cause the elevation of blood pressure (González-Núñez et al., 2000). The critical role of 20-HETE in the regulation of renal function and vascular tone makes it participate in the development of HTN and cardiovascular diseases.

Carnitines – According to the researches, 13 kinds of carnitines were the biomarkers of DM (Kappel et al., 2017). It transported long-chain fatty acids to the mitochondria and then to be oxidized to produce energy. In the cardiac muscle and skeletal, carnitine utilizes fatty acids as fuel. Insulin resistance is associated with oxidative defects in muscle fatty acids and played a crucial part in the development of type 2 DM (Mingrone, 2004). Studies also have shown that intravenous L-carnitine supplementation reduces insulin sensitivity in the distal muscles of diabetic patients (Gaetano et al., 1999). Finding from the study shows that carnitine lowers diastolic blood pressure in adults (Askarpour et al., 2019). L-carnitine and propionyl-L-carnitine can reduce the species of reactive oxygen species in HTN and increase the involvement of nitric oxide (NO) in endothelium-dependent relaxation (Bueno et al., 2005). The action of carnitine-dependent enzymes produces acetyl-CoA through the β-oxidation pathway, which modulates the intramitochondrial acetyl-CoA/CoA ratio affecting glucose oxidation (Calvani et al., 2000). An affected carbohydrate and lipid metabolism are verified in type 2 DM and HTN. The dysregulated fatty acid metabolism, along with tissue lipid accumulation, is associated with the development of HTN in type 2 DM. As the dominant role of carnitines in the balance of carbohydrate and fatty acid metabolism, it is likely to be a potential adjuvant in the treatment of type 2 DM.

BAs – BAs are steroid acids found in the bile of mammals predominantly. Primary BAs (cholic acid and chenodeoxycholic acid) are synthesized in the liver. Intestinal bacterial converts primary BAs into secondary BAs (Russell, 2003). In humans, taurocholic acid and glycocholic acid and taurochenodeoxycholic acid and glycochenodeoxycholic acid are the main bile salts in bile, with approximately equal concentrations (Hofmann, 1999). The level of taurine-conjugated BAs is elevated in type 2 diabetes (Liu et al., 2013), and the ratio of 12-α OH BAs has been reported to relative to insulin resistance (Hausler et al., 2013). Acarbose increases the ratio of primary BAs and secondary BAs. Uncombined BAs have also been proved to be elevated in type 2 DM patients at the beginning of treatment (Gu et al., 2017). Taurine is higher in DM and HTN groups (Fujiwara et al., 2005; Yokoi et al., 2015). An enhancement of creatinine was occurred in HTN, while it decreases in type 2 DM (Grapov et al., 2015). The supplementation with cholic acid and activation of the G protein-coupled bile acid receptor can significantly reduce the blood pressure (Shi et al., 2021).

Gut microbiome-related metabolites – In recent years, the relationship between the complexity and diversity of the gut microbiota and host diseases has been demonstrated (Weiss and Hennet, 2017; Li et al., 2018b). The gut microbiota constantly communicates hypotheses were brain-gut-microbiome axis (Mahony et al., 2015), brain-gut-kidney axis (Yang et al., 2018), and gut-liver axis (Tripathi et al., 2018). It produces several metabolites and accumulated in the bloodstream (Nicholson et al., 2012). High levels of AAAs, such as AA-phenylalanine, tyrosine, and glycine, occurred (Neis et al., 2015). Antidiabetic medication Acarbose was proved to alter plasma and fecal BAs composition *via* the mediate gut microbiota. The relative abundances of Lactobacillus and Bifidobacterium in the gut microbiota increased, while Bacteroides reduced. Moderating the relative abundance of microbial genes is related to bile acid metabolism (Gu et al., 2017). Studies have shown that the negative correlation between the hippurate reflects the physiological relationship with gut microbial (Holmes et al., 2008). Minocycline can cure resistant HTN patients because of its potent antihypertensive effect on the gut microbiota (Qi et al., 2015). Gut microbial composition research found that hypertensive rats having reduced taxa richness and altered microbial composition. The Firmicutes to Bacteroidetes ratio was higher in the hypertensive rats (Yang et al., 2015). Salt-sensitive rats showed higher blood pressure rather than salt-resistant rats, which was related to the elevated plasma levels of the short-chain fatty acids (SCFAs). It suggested gut microbiota-derived SCFAs produce the endocrine hormones. SCFAs, as a source of energy for colonocytes and bacterial communities, help maintain gut barrier function by inhibiting pathogenic microorganisms and reducing luminal pH. Host-signaling mechanisms, such as the G-protein-coupled receptors (GPR) 41 and olfactory receptor 78 (Olfr78), can modulate blood pressure (Tang and Hazen, 2017). Trimethylamine-N-oxide (TMAO), amino acids, and creatine related to energy metabolism were proved to contribute to HTN (Mell et al., 2015). TMAO was proved to be a strong predictor of coronary artery disease risk. Mechanistically, gut microbes transformed trimethylamine-containing nutrients, such as carnitine, phosphatidylcholine, and choline to TMAO, which leads to atherogenesis and thrombosis. In mice, increasing TMAO level decreased reverse cholesterol transport and altered bile acid transport, composition and pool size (Koeth et al., 2013). Meanwhile, TMAO modulated stimulus-dependent calcium mobilization in platelets, enhancing platelet responsiveness (Zhu et al., 2016). Accompanying metabolism functional changes, gut dysbiosis has been implicated in the pathogenesis ranging from insulin resistance to atherosclerosis and thrombosis.

What is noteworthy is that the biggest percentage of the total disturbed metabolites share a common biological function: provide energy. Besides amino acids, energy-related metabolites, lipids, and fatty acids, carnitines play the regulation role of the fatty acids and glucose metabolism in physiological and pathological conditions. A decreased ratio can relieve the transfer of acetyl groups from acetyl-CoA to carnitine, forming acetylcarnitine, a reaction catalyzed by carnitine acetyl-transferase. This activity of L-carnitine in the modulation of the intramitochondrial acetyl-CoA/CoA ratio affects glucose oxidation (Calvani et al., 2000). Other circulating substrates, such as ketones or BCAAs, may become an alternative source of energy.

## The Connection of Mechanism Underlying Metabolic Perturbation Between Type 2 DM and HTN

**Figure 2:**
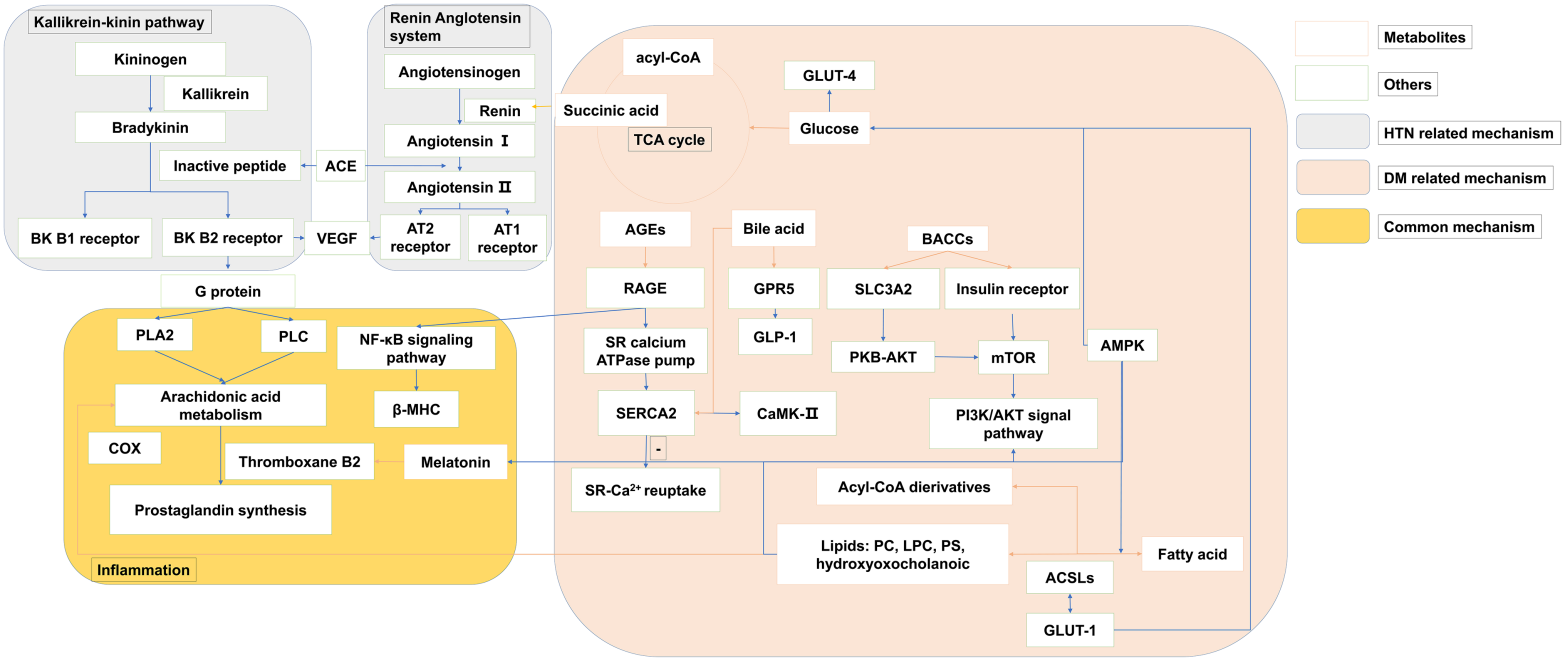
The overview of metabolites related common mechanisms of type 2 DM and HTN. *The pink* panel summarized the metabolites mechanism related to type 2 DM. The *gray panel* consists of the kallikrein-kinin pathway and the renin-angiotensin system, which are the main progress of HTN. *The yellow panel* presented the common metabolic mechanism of both type 2 DM and HTN. *Orange row* arrows the association of metabolites and biomolecules.

The development of metabolomics has facilitated the pathophysiology discovery of DM and HTN. The biomarker identified to reveal the mechanism of diseases *via* early changes highlights pathways and targets. Integration of the metabolism field with other disciplines, such as endocrinology and immunology, hints the metabolic alterations and substrate utilization. Here, as shown in Figure 2, we associated the biological function of metabolites with the mechanism of DM and HTN. DM and HTN are frequent comorbid conditions that may share underlying metabolic pathways, including the sympathetic nervous and renin-angiotensin-aldosterone system, insulin resistance, and inflammation processes (Schutta, 2007; Cheung and Li, 2012).

### Role of Metabolic Perturbations in HTN

A variety of disorders have been identified in HTN, including the renin-angiotensin system, the autonomic nervous system, and the immune system (as shown in the *gray* panel in Figure 2). Changes in the autonomic nervous system associated with increases in peripheral and neuroinflammation are associated with the pathogenesis of drug resistance to HTN. Recent evidence suggests that the increased peripheral and neuronal inflammation of HTN is due to the effect of autonomic nerves on bone marrow activity (Zubcevic et al., 2014). The SCFA olfactory Olf78 is also expressed in the kidneys, where it regulates blood pressure. Elevated FFAs may alter membrane fluidity by altering Na^+^-K^+^ ATPase pump, Na^+^, K^+^, and Ca^2+^ currents, membrane phospholipids structure, and intracellular Na^+^ and Ca^2+^ levels. This is the mechanism that increased the vascular muscle tension and consequently HTN (Ordway et al., 1991).

Emerging evidence suggested that gut microbiota plays a crucial part in the development of HTN. This effect was thought to be mainly due to the SCFAs (Holmes et al., 2008). The phylum *Bacteroidetes* and family *Veillonellaceae* were proved to be more abundant in salt-sensitive than in salt-resistant rats (Mell et al., 2015).

### Role of Metabolic Perturbations in DM

As shown in the *pink* panel in Figure 2, the increased level of BCAAs was improved to make an influence in insulin sensitivity *via* the mammalian target of rapamycin complex (mTORC) and target ribosomal protein S6 kinase 1 (S6K1) in the downstream (O’Connell, 2013). The effector of mTOR, S6K1, is sensitive to both AA and insulin. Phosphatidylinositol 3-kinase (PI3K)/Akt signaling can downregulate autophagy by inhibiting the mTOR, and the changes in autophagy were associated with reduced PI3K/Akt signaling in insulin resistance (Bugger and Abel, 2014). The numbers of metabolites separating the DM groups were associated with mitochondria. The centrality of the mitochondrial function in catabolic and metabolic diseases of BCAA has been noted previously (Newgard, 2012). BCAA may damage mitochondrial oxidation of glucose and lipids, which can lead to mitochondrial stress and impaired insulin secretion and action. Impaired mitochondrial function in DM could decrease the capacity of the mitochondria to decompose BCAAs, resulting in elevated levels of BCAAs and BCKAs. The dysfunction of educed mitochondrial biogenesis was rescued by augmentation of adenosine 5′monophosphate-activated protein kinase (AMPK) activity (Dugan et al., 2013). The studies showed that the peptide hormone is produced by adipose cells, such as adiponectin/Acre 30 and retinol-binding protein-4 (RBP4). They also produce resistin and proinflammatory cytokines, such as interleukin-6 (IL-6) and tumor necrosis factor-α (TNF-α). Several BA species have been recognized as modulators of energy metabolism by activation of nuclear receptors, such as G-protein-coupled BA receptor (TGR5) and farnesoid X receptor (FXR) (Thomas et al., 2008). The activation of FXR induces fibroblasts to grow in the intestine. Although the mechanisms are not yet fully understood, the release of glucagon-like peptide 1 (GLP-1) and peptide YY (PYY), as well as changes in BA metabolism and enhancing signaling *via* FXR, is thought to play a role. TGR5 is necessary to improve glucose metabolism, which may be related to DM (Tremaroli et al., 2015). SCFAs can stimulate GPR 41 and 43, and these are expressed in the renal vasculature. These effects include several processes such as inflammation and intestinal endocrine regulation (Samuel et al., 2008). However, the balance across these G-protein-coupled receptor activities is complex and likely to be dynamic (Pluznick, 2013). SCFAs can also trigger GPR41 and GPR43 to secrete GLP-1, which plays a substantial role in pancreatic function and insulin release. GPR41 induces intestinal endocrine hormone PYY expression in the epithelial L cells through increasing dietary energy gain (Samuel et al., 2008). Adipose cells also produce other peptide hormones, including retinol-binding protein RBP4 and adiponectin/Acrp 30). Fat-specific glucose transporter 4 (GLUT4) knockout mice showed elevated RBP4 levels (Yang et al., 2005). The expression of monocyte chemotactic protein-1 (MCP1) and IκB kinase catalyzes injury and infection of subunit-β (IKKβ) recruitment sites in the adipocyte. It may be a mechanism for increased inflammatory signals during the development of DM (Arkan et al., 2005; Chen et al., 2005).

Recent studies show that individual bacteria concerned with insulin resistance have the same phenotype for recipient mice when transferred to normal, specific, and non-pathogenic mice (Pedersen et al., 2016; Liu et al., 2017b). Amino acids act on mTOR receptor regulating pathways and physiological processes, like insulin. Gut microbiota can regulate TGR5 signal-generating receptor agonists and FXR signal metabolism antagonists, such as tauro-β-muricholic acid (Sayin et al., 2013). TGR5 is required for improved metabolism of glucose (Tremaroli et al., 2015). Propionate SCFs stimulate GLP1 and PYY hormone release in the primary gut culture of mice through the FFA receptor 2 (FFAR2) dependent mechanism (Tolhurst et al., 2012). Gut microbiota induces PYY in an FFAR3-dependent manner (Psichas et al., 2015).

### Role of Metabolic Perturbations in DM and HTN

As shown in Figure 2 (*yellow* panel), both of the metabolic perturbation mechanisms of DM and HTN link to the inflammation. Subjects with DM had elevated plasma FFA levels, which played a detrimental role in the pathogenesis of HTN. FFAs lead to multiple mechanisms, including the activation of the renin-angiotensin system, impaired insulin signaling and NO production, oxidative stress, inflammation, and apoptosis in the endothelial cells, which make an influence in the DM and HTN co-exist (Ghosh et al., 2017). Melatonin has a special place in the prevention and treatment of metabolic syndromes, such as DM and HTN (Cardinali et al., 2011). It has an anti-inflammatory property, partly for its role as a metabolic regulator. Melatonin’s resistance to inflammation occurs in the correction of metabolic disorders, which prevent insulin resistance. Impaired serine phosphorylation of insulin receptor substrate 1 (IRS-1) and the subsequent upregulation of IRS-1 expression may be crucial (She et al., 2014). *Via* the activation of CREB-PGC-1α pathway, melatonin could prevent insulin resistance and mitochondrial dysfunction (Teodoro et al., 2014). Both melatonin and melatonergic agonists counteract the blockade of this critical step in insulin signal (She et al., 2009; Quan et al., 2015). Regeneration and pancreatic β-cells lead to decreased blood glucose after melatonin treatment in the diabetic rats model (KanterEmail et al., 2006). Melatonin treatment improves insulin sensitivity and lipid metabolism in type 2 diabetic rats (R.C. Mazepa et al., 1999; Shieh et al., 2009). The inhibition effect of melatonin on platelet aggregation and thromboxane B2 was also proved.

## Type 2 DM-Associated Metabolic Perturbations Potentially Involved in the Pathogenesis of HTN

**Figure 3:**
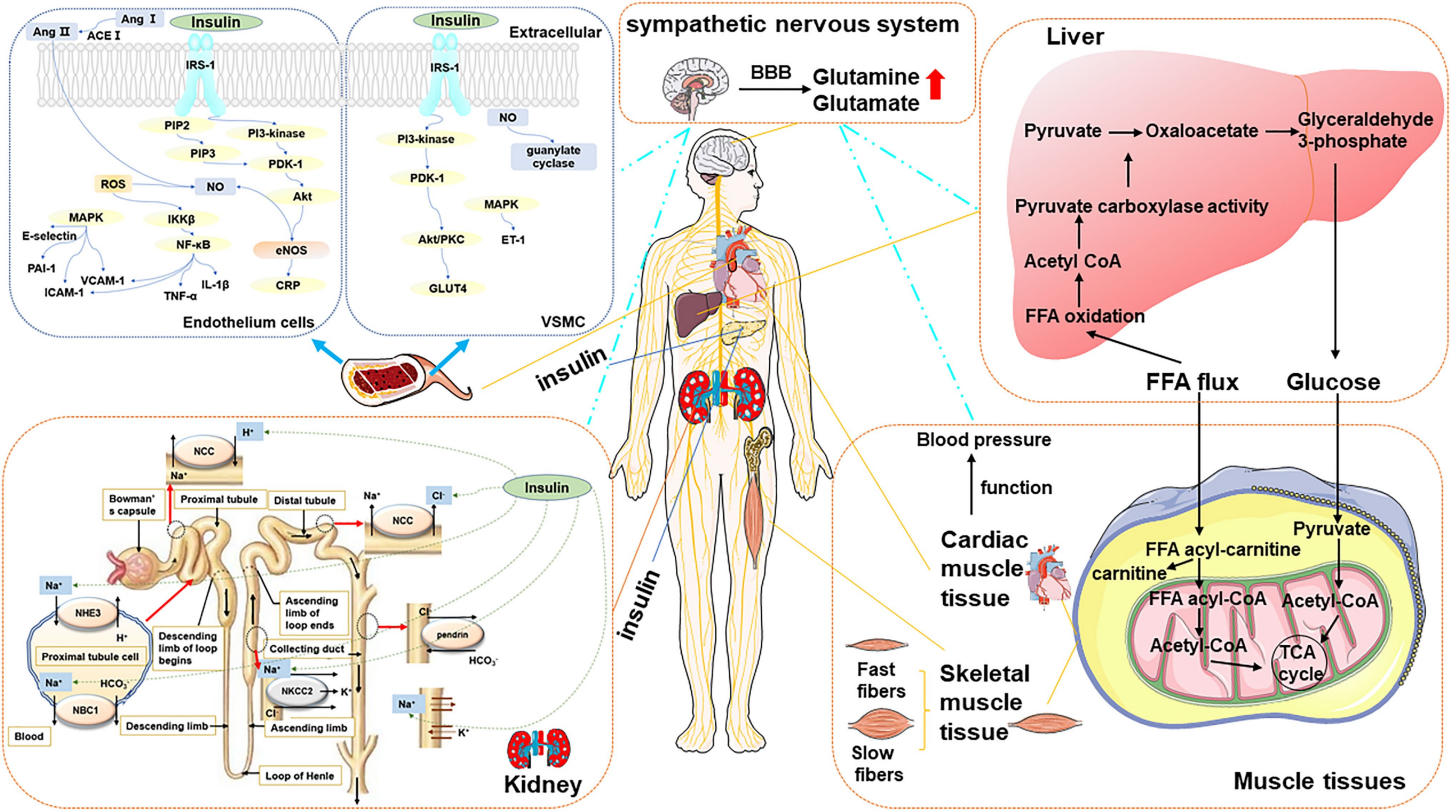
The overview of type 2 DM-associated metabolic perturbations potentially involved in the pathogenesis of HTN. *Blood vessel The left panel* presented the mechanisms of HTN in DM in the endothelium cell. The mechanism started with IRS triggered activation of PI3K and the conversion from PIP_2_ to PIP_3_. It allowed PDK-1 to access, leading to Akt activation. Akt increased eNOS activity and stimulated the production of NO. Meanwhile, Ang II and ROS are another way to produce NO. MAPK-dependent insulin signaling pathways tend to promote pro-HTN insulin metabolite. It inhibits PAI-1, E-selection, ICAM, and VCAM. *The right panel* presented the mechanism of HTN in DM in VSMC. The increased blood flow further facilitates glucose uptake by provoking insulin-responsive GLUT4 translocation in PI3K-dependent metabolic actions. MAPK-dependent insulin signaling pathways tend to promote ET-1. The production of NO is related to guanylate cyclase. *Kidney* The sodium-chloride cotransporter (NCC) has the function of reabsorbing sodium and chloride ions from the tubular fluid. Insulin promotes the resorption of chloride ions. Insulin stimulates the NHE3 on the apical membrane and upregulates the NBC1 on the basolateral membrane. In the thick ascending limb of Henle, insulin stimulates NaCl reabsorption by activating NKCC2 and Na-K-ATPase. The sodium and bicarbonate were reabsorbed. Insulin can also stimulate the Na-K-ATPase in the proximal tubule in the proximal tubule. *Liver and muscular tissue* FFA is the major oxidation fuel for muscular tissue, such as cardiac muscle tissue and skeletal muscle tissue, while glucose provides the remaining need. The transformation of cardiac function metabolites affects the function of the heart, which is closely related to blood pressure. The balance of FFA and glucose results in the transformation of two muscle types: slow-twitch myofibers and fast-twitch fibers. *Sympathetic nervous system* It controls peripheral organs, including the liver, skeletal muscle, pancreas, vascular and cardiovascular system resulting in glucose disorder, insulin resistance, insulin secretion impairment, and blood pressure increase, which are predicted to chronically contribute to the development of DM and HTN.

Perturbated metabolites exert several physiological responses either directly through the activation of powerful humoral systems, indirectly stimulation localized target organs, or *via* postganglionic sympathetic nerves. The actions and interactions of insulin, angiotensin II, inflammation, vascular dysfunction, or central nervous systems lead to the pathogenesis and progression of HTN. Here, we pictured the overview of physiological processes in Figure 3.

### Insulin Sensitivity in Cells and Tissue

Reduced sensitivity to insulin in tissue is a characteristic of multiple pathological conditions (Du et al., 2006). HTN in subjects with DM was significantly related to insulin resistance (Rewers et al., 2004). Acute hyperglycemia consistently impairs endothelial function in type 2 DM patients (Wallis et al., 2005). Reducing glucose levels may inhibit vascular changes leading to HTN (The DCCT Research Group, 1988; Purnell et al., 1998).

Insulin metabolism is often mediated by insulin signaling. PI3K/Akt pathway causes atherothrombosis *via* multiple mechanisms. On the one hand, it produces several beneficial molecules. On the other hand, it inhibits plasminogen activator inhibitor type 1 (PAI-1), E-selection, intercellular adhesion molecule (ICAM), and vascular cell adhesion molecule (VCAM). The increased expression level of VCAM1 and ICAM1 and inflammatory cytokines, like interleukin IL6, IL18, IL1β, TNF α, and TGF-β, lead to myocyte inflammation in the DM and HTN (Rajesh et al., 2012). Glucotoxicity, lipotoxicity, and inflammation impair the PI3K-dependent insulin signaling pathway, which constructs the combination of DM and HTN (Muniyappa et al., 2008). PI3K-dependent insulin signaling pathways regulate vasodilator and insulin metabolite in skeletal muscle and vascular endothelium. MAPK-dependent insulin signaling pathways tend to promote pro-HTN insulin metabolite. Insulin resistance, hyperglycemia, oxidative stress, and attenuate inflammation and renin-angiotensin process in endothelial smooth muscle and kidney issues are predicted to make a simultaneous beneficial influence in the co-existed DM and HTN.

In vein endothelial and endothelial cells – One of the important roles of insulin is to accelerate the production of the effective vasodilator factor NO by endothelial cells (Vincent et al., 2003; Muniyappa et al., 2007). The binding of insulin binding to its receptor (a receptor tyrosine kinase) causes phosphorylation of IRS-1, which can activate PI3K. The lipid products of PI3K [PI-3,4,5-triphosphate (PIP3)] can stimulate the phosphorylation and activation of PDK1, which in turn phosphorylates and activates Akt. Akt increased the eNOS activity and stimulated the production of NO, which is calcium-independent (Montagnani et al., 2001). NO inhibits the production of ROS, which leads to endothelial dysfunction and promotes the development of atherosclerosis (Muniyappa et al., 2008). ET-1, as a potent vasoconstrictor, is stimulated by insulin and plays a crucial role in endothelial dysfunction and contributes to the development of HTN, using the MAPK-dependent signaling pathway (Marasciulo et al., 2006). And the increased expression level of PAI-1. ICAM-1, VCAM-1, and E-selectin may contribute to accelerated atherosclerosis in insulin-resistant conditions (Bonetti et al., 2003). Insulin stimulates the expression of VCAM-1, PAI-1, and E-selectin in endothelial cells through the MAPK-dependent pathway (Montagnani et al., 2002; Mukai et al., 2007). The suppression of PI3K or Akt174 increased the insulin-induced PAI-1 and adhesion molecule expression.

When endothelial cells are exposed to high concentrations of glucose, insulin-stimulated Akt and eNOS activation is significantly reduced (Du et al., 2001; Wang et al., 2006). Hyperglycemia is also believed to be associated with impaired glucose tolerance. It induces an increasing level of ROS production, post-translational O-GlcNacylation, PKC activity, and advanced glycation end products (AGEs), which is known to specifically inhibit the PI3K/Akt/eNOS pathway. Diabetes causes insulin resistance and endothelial dysfunction by increasing oxidative stress, and formatting AGEs (Du et al., 2001; Muniyappa et al., 2007). Compared with the harmful effects of the PI3K/Akt/eNOS pathway, hyperglycemia can enhance the secretion of endothelial endothelin (ET-1), thereby changing the balance between NO and ET-1, which is beneficial to endothelial dysfunction (Muniyappa et al., 2008). *In vivo*, acute intravenous glucosamine causes metabolic insulin resistance and impairs insulin-mediated increase in femoral arterial blood flow and capillary replacement (Wallis et al., 2005).

Elevated circulating markers of inflammation characterize insulin resistance and endothelial dysfunction. The most widely studied proinflammatory cytokine related to insulin resistance is IKKβ, TNF-α, and C-Jun N-terminal kinase (JNK) (Kim et al., 2005). IL-1β receptor-associated kinase was activated by TNF-α, which stimulated serine phosphorylation of IRS-1/2 directly or indirectly. It compounds the decreasing activation of PI3K/Akt/eNOS in endothelial cells (Zhang et al., 2003; Anderson et al., 2004; Eringa et al., 2004; Gustafson et al., 2007). TNF-α can also increase ET-1 secretion in a MAPK-dependent fashion (Sury et al., 2006). Another cytokine, IL-6, was elevated and can inhibit increased eNOS activity and increased NO production stimulated by insulin in endothelial cells (Andreozzi et al., 2007). C-reactive protein (CRP), which served as a marker of inflammation, was proved to inhibit insulin-induced NO production in endothelial cells by inhibiting the PI3K/Akt/eNOS pathway (Schwartz et al., 2007; Xu et al., 2007).

In vascular smooth muscle and vascular smooth muscle cells (VSMC) – Endothelial NO diffuses into adjacent VSMC, where it stimulates guanosine cyclase. The elevated levels of cGMP can give rise to vasorelaxation. NO can also modulate vascular tone by reducing monocyte adhesion, increasing the production of proinflammatory cytokines, attenuating the expression of vascular cell adhesion molecules, limiting the recruitment of leukocytes, inhibiting VSMC, avoiding platelet aggregation, and opposing apoptosis (Herman and Moncada, 2005). The increased blood flows further facilitate the glucose uptake by provoking insulin-responsive GLUT4 in PI3K-dependent metabolic actions (Zeng and Quon, 1996; Vincent et al., 2004). In human cells, three IRS subtypes (IRS-1, -2, and -4) have been identified to have a distinct role based on cell type and metabolic status. The function of IRS-1 is related to the insulin secretion mechanism, which plays a vital role in skeletal muscle (Taguchi and White, 2008). Animal models showed retarded growth of IRS-1 knockout mice especially skeletal muscle (Withers et al., 1998). Glucosamine can impair insulin and stimulate the uptake of glucose in skeletal muscle and NO production. It can also increase the blood flow in femoral arterial and capillary recruitment (Wallis et al., 2005).

In the kidney – Reduced insulin sensitivity leads to compensatory hyperinsulinemia. Maintaining normal blood sugar, glucose is taken up by skeletal muscle and adipose cells. Compensatory hyperinsulinemia with insulin resistance reinforced the salt absorption of the proximal tubule, which may lead to salt overload and HTN (Soleimani, 2015). The sodium-chloride cotransporter (NCC) has the function of reabsorbing sodium and chloride ions from the tubular fluid. Insulin promotes the resorption of chloride ions. The effect of insulin on IRS1-mediated adipocyte glucose uptake is severely attenuated, and the effect on IRS2-mediated proximal renal tubular salt reabsorption remains (Zheng et al., 2005). The kidney tubules are significant for maintaining vascular flow and blood pressure because of the sodium reabsorption regulation. Insulin was proved to promote salt reabsorption. Insulin stimulates the Na^+^/H^+^ exchanger type 3 (NHE3) on the apical membrane and upregulates the sodium-bicarbonate cotransporter (NBC1) on the basolateral membrane. The sodium and bicarbonate were reabsorbed. Insulin can also stimulate the Na-K-ATPase in the proximal tubule in the proximal tubule (Landsberg et al., 2013). In the thick ascending limb of Henle, insulin stimulates NaCl reabsorption by activating Na-K-2Cl cotransporters (NKCC2) and Na-K-ATPase (Horita et al., 2011). Insulin enhanced the epithelial sodium channel density in the distal nephron and the connecting tubule membrane (Landsberg et al., 2013). The accumulated reports showed that the stimulatory effect of insulin on with-no-lysine kinases (WNK), suggesting that sodium increases sodium reabsorption through sodium-chloride cotransporter in the distal nephron (Landsberg et al., 2013).

### The Activation of Angiotensin II

DM-induced hyperglycemia can cause HTN by activating Angiotensin II (Ang II). Ang II is involved in the pathogenesis of insulin resistance through the induction of key signaling elements of the insulin AKT (also known as protein kinase B) pathway (Andreozzi et al., 2004). It also serves as an essential vasoconstrictor hormone in the renin-angiotensin system (RAS), where RAS is activated (Kobori et al., 2007). These findings suggested that the cross-action of insulin AKT and Ang II signaling pathways plays a significant role in the co-occurrence of DM and HTN. Ang II inhibits insulin metabolism signals and promotes insulin resistance by activating the mTOR/S6 kinase 1 (S6K1)-mediated IRS-1 serine phosphorylation (Pulakat et al., 2011). Ang II reduces glucose uptake by inhibiting IRS1 phosphorylation, inhibiting PI3-K function and reducing GLUT-4 to interfere with the insulin pathway (Manrique et al., 2009). Moreover, Ang II hinders the bioavailability of NO through NADPH oxidase activation and ROS generation, which impeding endothelium. And it can also enhance NF-κB, which in turn promotes the production of TNF-α and IL-6 and adhesion molecule VCAM-1, regulating inflammation (Pueyo et al., 2000).

### The Activation of the Sympathetic Nervous System

The activation of the sympathetic nervous system exerts a number of physiological responses either directly through stimulation of postganglionic sympathetic nerves localized in target organs or indirectly through the activation of powerful humoral systems. The importance of sympathetic tone is readily acknowledged for blood pressure regulation and type 2 DM (Thorp and Schlaich, 2015). The sympathetic nervous system control over peripheral organs, including the liver, skeletal muscle, pancreas, and cardiovascular system, is perturbed, resulting in glucose disorder, insulin resistance, insulin secretion impairment, and blood pressure increase, which are predicted to chronically contribute to the development of DM and HTN. Otherwise, the RAS and inflammation activated by metabolites or insulin are demonstrated to increase the central sympathetic nervous system activity to elevate blood pressure.

Glutamate and glutamine, as common metabolic perturbations of both DM and HTN, are listed in Table 1. Previous research indicated that glutamate could penetrate the blood–brain barrier, excite the presympathetic neurons within the paraventricular nucleus, and increase the sympathetic outflow (Li et al., 2006). Glutamine abounds in the central nervous system, and its interstitial and cerebrospinal fluid concentration are at least one the order of magnitude higher than of any other amino acid. It is a major excitatory neurotransmitter in the vertebrate nervous system, and glutamatergic synaptic inputs innervate the presympathetic neurons located in the autonomic nucleus in the brain stem and hypothalamus. The effects of metabolites on brain regions involved in insulin and blood pressure regulation are a mechanism of DM that resulted in the pathogenesis of HTN.

### The Energy Reprograming

It found that the commonality of DM and HTN among metabolites is limited. How they are different or more interestingly amalgamated in both disease phenotypes, which leads to an intermediate or tertiary state, should be explored. After summarizing the metabolic distribution in DM and HTN, amino acids, energy-related metabolites, lipids, and fatty acids were revealed to be made up the biggest percentage of the total disturbed metabolites and share a common biological function: provide energy. Here, we discussed the energy-provide metabolites transformation in the cardiac muscle and skeletal muscles.

Cardiac muscle – The transformation of cardiac function metabolites seems to disturb much more metabolites and affect the pathogenesis of HTN in type 2 DM. In the normal heart, carbohydrate and FFA oxidation mainly contribute to energy production. While type 2 DM dysregulated cardiac FFA oxidation and impaired glucose oxidation/uptake, other circulating substrates, such as ketones or BACCs, may become an alternative source of energy, which injured the heart and cause high blood pressure. Bedi et al. (2016) recently demonstrated that in the severely failing heart increased the utilization of ketone. Although the role of BCAA is less clear because of the diminishment of BCAA catabolism in heart failure, ketones and BCAAs can directly influence cardiac signaling processes. It may potentially exhibit additional beneficial effects on the heart. In the recent EMPA-REG OUTCOME trial, treatment with empagliflozin, it was observed the reduction of cardiovascular mortality and hospitalization for heart failure in patients with type 2 DM. The research that investigated the effect of empagliflozin on metabolism showed that it reduced the glucose and other sugars in the serum and degraded FFAs, amino acids, and ketone with increased levels of acetyl- and propionyl carnitine.

Skeletal muscles – Similar to the heart muscle, skeletal muscles play key roles in the regulation of systemic energy homeostasis in metabolic responses to physical activity. Excess triglycerides, FFAs, and glucose, coupled with physical inactivity, perturb the metabolism in skeletal muscle. Skeletal muscle made an influence on energy homeostasis by changing the composition of slow and fast-twitch fiber types muscles, which differ in the composition of contractile proteins, oxidative capacity, and substrate preference for ATP production (Baskin et al., 2015). Fast-twitch fibers have a higher fatigability, higher strength of contraction, lower oxidative capacity, and a preference for glucose as a substrate for ATP production through anaerobic glycolysis. Slow-twitch myofibers have a high oxidative capacity and prefer fatty acids as substrate for ATP production, while fast-twitch fibers have a lower oxidative capacity and prefer glucose.

Muscle profoundly impacts systemic energy consumption. In the pathological state of DM, the energy reprogramming changes the metabolic function of the heart and then changes the cardiac output which affects blood pressure directly.

## Conclusion and Perspectives

Type 2 DM is known to be associated with HTN. The widespread of type 2 DM also poses many problems. The presence of type 2 DM increases the risk of HTN. When combined with DM, HTN has been shown to predict and promote increased risk for cardiovascular diseases, which is the leading cause of death in patients with DM. Metabolites reflect both the upstream input from the environment and the downstream output of the genome, making it possible to explore the interaction between genes and the environment. Focusing on the application of metabolomics seems to contribute to reveal the root cause of type 2 DM. Knowledge of the pathophysiological derangements involved in the occurrence of type 2 DM and HTN is critical for successful prevention and control solutions. In this review, metabolites perturbation and the underlying metabolic mechanism were summarized. Among the metabolites, amino acid, lipid metabolites, carnitines, bile acids, and other metabolites were elaborated. And the related metabolites resulted in insulin resistance, increased tissue inflammatory, ROS production, endothelial dysfunction, the distribution of the renin-angiotensin system, the activation of the sympathetic nervous system, and the energy reprograming.

There were several limitations in the present review. First, the prior mechanism studies that are relevant to the thesis might be limited because of the scope of the HTN topic. The complex influence factors are involved in the endocrine system, nervous system, and immune system. Direct influences and feedback interaction conveyed by the bloodstream from one tissue to another exacerbate the complexity of the problem. The deep mechanism researches should continue to be followed up in the future. Second, the current review is focused on how perturbated metabolites in diabetes mellitus affect the pathogenesis of hypertension. A contextual focus on *vice-versa* relations may need in-depth further exploration and contextual consideration.

With the development of informatics, integration of orthogonal biological approaches, and analytical technologies, it is of possibility to expand the application of metabolomics to understand the systemic function of metabolites. It seems to be promising to elaborate the mechanism in HTN in type 2 DM by regarding the metabolites as the beginning of the biological process and reveal the active role (Rinschen et al., 2019). With the review, the mechanism of HTN in type 2 DM may be investigated by gut microbial, inflammation, and energy metabolism processes.

### Gut Microbial in HTN and Type 2 DM

Intestinal microbial populations contribute to energy collection, host metabolism, and disturbances in these population balances affect inflammation, glucose metabolic changes, and energy metabolomics. Furthermore, through diligent research efforts in gut microbial metabolites, the underlying mechanisms of type 2 DM and HTN are beginning to be elucidated. Microbial influenced metabolites were proved to link the microbiota and host blood pressure regulation (Shi et al., 2021). Microbial gene was proved to facilitate metabolites conversion by functional and genetic engineering studies. The microbial *porA* gene promotes phenylalanine change to phenylacetic acid, with the host generation fostering platelet responsiveness and thrombosis potential (Nemet et al., 2020). The *Casp1*^−/−^ microbiota reduced microbiota-derived anti-inflammatory SCFAs (Brandsma et al., 2019). The gut microbiota also proved to regulate blood glucose *via* modulating the enteric neurons (Matheis et al., 2020).

### Inflammation in HTN With Type 2 DM

In this review, inflammation is proved to be a potential common mechanism of type 2 DM and HTN. It is widely acknowledged that inflammatory disorder is the main pathological process of many diseases (Hummasti and Hotamisligil, 2010). Indeed, subclinical chronic inflammation is a common feature in their natural course. The application and development of pro-resolution therapeutic strategies for treating inflammatory pathology could lead to the revolutionary management of human ailments. Fullerton and Gilroy (2016) highlighted strive to identify pathways that target diseases that involve inflammation. Age, as a risk factor of the inflammation, underlies type 2 DM and HTN (Yandrapalli et al., 2018). Elie Metchnikoff proposed that bacterial products activated phagocytes resulting in inflammation (Metchnikoff, 2003). Aging-associated microbiota was proved to promote inflammation and reversing age-related microbiota variation for reducing inflammation and the accompanying morbidity in recent research (Thevaranjan et al., 2017). And the enhancement of autophagy and the normalization of mitochondrial function can alleviate aging-associated inflammation (Bharath et al., 2020). Novel insights into the cellular processes especially the metabolic reprogramming of immune cells driving inflammation are more or less established (Pålsson-McDermott and O’Neill, 2020). Adaptive immune memory, macrophage iron handling, epigenetic regulation of immunity, and Th2 immunity can directly affect immune function and ultimately systemic metabolism (Caslin and Hasty, 2019). The mechanism by which DM and HTN may synergistically induce macrophage metabolic dysfunction, especially during cardiac remodeling, is not fully understood (Mouton et al., 2020). It will provoke a re-think of chronic inflammation in HTN and type 2 DM therapeutically.

### Energy Metabolism Switch of the Immune System in HTN With Type 2 DM

The idea that cellular metabolism affects immune function is currently being pursued. The physical activities and cellular functions related to energy were decreased owing to unmatched energy demand and supply. In return, the metabolic shift was accelerated and forming a vicious cycle. Reprogramming of energy metabolism causes a shift in energy metabolism from oxidative metabolism to glycolysis, which promotes the prevalence of aging-associated diseases, such as type 2 DM and HTN (Feng et al., 2016). These findings provide a reference for future research on the pathogenesis of type 2 DM and diabetic complications.

## Author Contributions

ZN and ZS wrote the paper. CW, SP, and XW revised the paper. ZL and AL designed the outline of the paper. All authors contributed to the article and approved the submitted version.

## Conflict of Interest

The authors declare that the research was conducted in the absence of any commercial or financial relationships that could be construed as a potential conflict of interest.

## Publisher’s Note

All claims expressed in this article are solely those of the authors and do not necessarily represent those of their affiliated organizations, or those of the publisher, the editors and the reviewers. Any product that may be evaluated in this article, or claim that may be made by its manufacturer, is not guaranteed or endorsed by the publisher.

## Figures and Tables

**Table:** 

DM	Diabetes mellitus
HTN	Hypertension
LC-MS	Liquid chromatography-mass spectrometry;
GC-MS	Gas chromatography-mass spectrometry
NMR	Nuclear magnetic resonance
GC	Gas chromatography
LC	Liquid chromatography
PCA	Principal component analysis
PLS	Partial least squares
OPLS-DA	Orthogonal partial least square discriminant analysis
OSC	Orthogonal signal correction
LASSO	Least absolute shrinkage and selection operator
BCAAs	Branched-chain amino acids
AAAs	Aromatic amino acids
TCA	Tricarboxylic acid
PC	Phosphatidylcholine
LPC	Lysophosphatidylcholine
PE	Phosphatidylethanolamine
DAG	Diacylglycerol
SM	Sphingomyelins
Cer	Ceramide
PA	Phosphatidic acid
LPA	Lysophosphatidic acid
HODEs	Hydroxyoctadecadienoic acids
HETE	Hydroxyeicosatetraenoic acid
HPETE	Hydroxyeicosatetraenoic acid
BAs	Bile acids
DMG	Dimethylglycine
BHMT	Methyltransferase
DMGDH	Methyltransferase dehydrogenase
FFAs	Free fatty acids
LC-CoAs	Long-chain acyl CoAs
ER	Endoplasmic reticulum
NO	Nitric oxide
SCFAs	Short-chain fatty acids
GPR	G-Protein-coupled receptors
Olfr78	Olfactory receptor 78
TMAO	Trimethylamine-N-oxide
mTORC	The mammalian target of rapamycin complex
S6K1	S6 kinase 1
PI3K	Phosphatidylinositol 3-kinase
AMPK	Adenosine 5'-nomophosphate-activated protein kinase
RBP4	Retinol-binding protein-4
IL-6	Interleukin-6
TNF-α	Tumor necrosis factor-α
FXR	Farnesoid X receptor
GLP-1	Glucagon-like peptide 1
PYY	Peptide YY
GPR41	G-Protein-coupled receptors 41
GPR43	G-Protein-coupled receptors 43
GLUT4	Glucose transporter-4
MCP1	Monocyte chemotactic protein-1
IKKβ	IκB Kinase catalytic subunit-β
FFAR2	Free fatty acid receptor 2
IRS-1	Insulin receptor substrate 1
PAI-1	Plasminogen activator inhibitor type 1
ICAM-1	Intercellular adhesion molecule 1
VCAM	Vascular cell adhesion molecule
PDK1	Phosphoinositide-dependent protein kinase-1
ROS	Reactive oxygen species
PKC	Protein kinase C
ALSLs	Acyl-CoA synthetases
RAGE	Receptors for advanced glycation end
NF-κB	Nuclear factor kappa-B
β-MHC	β-Myosin heavy chain
PIP3	PI-3,4,5-Triphosphate
AGEs	Advanced glycation end products
ET-1	Endothelin 1
JNK	C-Jun N-terminal kinase
CRP	C-Reactive protein
VSMC	Vascular smooth muscle and smooth muscle cells
NHE3	Na^+^/H^+^ exchanger type 3
NBC1	Sodium-bicarbonate cotransporter
NKCC2	Na-K-2Cl Cotransporters
WNK	With-no-lysine kinases
RAS	Renin-angiotensin system
S6K1	S6 Kinase 1
